# Analysis of arrhythmia and its risk factors in patients with COVID-19

**DOI:** 10.1371/journal.pone.0336370

**Published:** 2025-11-25

**Authors:** Wenzhao Guan, Meihua Liu, Shuling Rong, Tianpei Liu, Xiaolin Wang

**Affiliations:** 1 Department of Stomatology, The Second Hospital of Shanxi Medical University, Taiyuan, China; 2 Academy of Medical Sciences, Shanxi Medical University, Taiyuan, China; 3 Department of Health Statistics, School of Public Health, Shanxi Medical University, Taiyuan, China; 4 Department of Cardiology, The second Hospital of Shanxi Medical University, Taiyuan, China; 5 The second Hospital of Shanxi Medical University, Taiyuan, China; Tokyo Women's Medical University: Tokyo Joshi Ika Daigaku, JAPAN

## Abstract

**Background:**

To investigate the incidence of arrhythmia in patients with coronavirus disease 2019 (COVID-19) and analyze its risk factors.

**Methods:**

This was a retrospective cross-sectional study that surveyed 324 COVID-19 patients admitted to the Second Hospital of Shanxi Medical University from 2020 to 2022. General data, vital signs, myocardial enzyme and imaging data of the patients were collected. The characteristics of COVID-19 patients with arrhythmia were analysed, and multivariate logistic regression was used to analyze the risk factors for arrhythmia in patients. The receiver operating characteristic (ROC) curve was plotted to evaluate the efficacy of the regression equation in predicting arrhythmia in COVID-19 patients.

**Results:**

Compared with COVID-19 patients without arrhythmia, those with arrhythmia were found to have significant differences in heart rate, prothrombin time (PT), activated partial thromboplastin time (APTT), blood glucose, uric acid, serum potassium, serum total cholesterol, high-sensitivity troponin I (hs-TnI), N-terminal pro B-type natriuretic peptide (NT-proBNP), aortic root diameter, left atrial diameter (LAD), left ventricular end-diastolic diameter (LVEDD), left ventricular ejection fraction (LVEF), fractional shortening (FS), end-systolic volume (ESV), and diabetes (all *P* < 0.05). Multivariate logistic regression analysis revealed that heart rate, PT, hs-TnI, ESV, serum potassium, blood glucose and diabetes were risk factors for arrhythmia in COVID-19 patients (all *P* < 0.05). ROC curve analysis showed that the area under the curve (AUC) was 0.773 (95%CI: 0.711-0.834, *P* < 0.001).

**Conclusion:**

Heart rate, PT, hs-TnI, ESV, serum potassium, blood glucose and diabetes are risk factors for arrhythmia in COVID-19 patients.

## 1. Introduction

A previously unknown pneumonia, was first reported in December 2019 in China [1], which then grew to become a global pandemic caused by the severe acute respiratory syndrome coronavirus 2 (SARS-CoV-2), commonly known as coronavirus disease 2019 (COVID-19) or simply novel coronavirus pneumonia. Initially considered a respiratory condition, COVID-19 has since revealed various extrapulmonary manifestations that were often overlooked in clinical settings. With the ongoing development of the COVID-19 pandemic, the increasing number of cases, and expanding clinical data, our understanding of COVID-19 continues to deepen. Researchers have identified an extensive range of complications associated with COVID-19. In addition to typical respiratory symptoms, patients have also experienced acute myocardial injury, arrhythmias and cardiac dysfunction. Arrhythmias are the common non-respiratory complication in COVID-19 patients [2]. During the pandemic, control efforts, factors related to the disease itself, psychological factors, and social factors led to a significant increase in the incidence of arrhythmias among COVID-19 patients. SARS-CoV-2 can directly cause myocardial damage, or it can induce arrhythmias through inflammatory responses [3]. Cardiac arrhythmia is a common type of disease that manifests in the cardiovascular system. When combined with COVID-19, the occurrence of adverse events such as cardiac arrest and sudden cardiac death significantly increases, posing a serious threat to the patient’s life [4]. In addition, studies have found that COVID-19 patients without a history of cardiovascular diseases may experience arrhythmia [5]. Recent research has confirmed the damaging effect of COVID-19 on the cardiovascular system. In light of the importance of cardiovascular diseases, especially arrhythmia, and their impact on the prognosis of COVID-19 patients, further investigation is required. This study aimed to investigate the incidence of arrhythmia in patients COVID-19, analyze its risk factors, and provide a basis for accurate treatment and management of patients with COVID-19. Enriching clinical medical personnel’s understanding of COVID-19’s pathophysiology and developing relevant treatments and solutions are of great significance.

## 2. Data and methods

### 2.1 Data collection

This retrospective study utilized data from medical records at the Second Hospital of Shanxi Medical University. The data collection period for research purposes spanned from July 10, 2024, to September 10, 2024. The study protocol was reviewed and approved by the Institutional Ethics Committee of the Second Hospital of Shanxi Medical University (Approval No. 2024-YX-298), which granted a waiver for informed consent due to the retrospective nature of the study. Throughout the entire process of data collection and analysis, the authors had no access to any information that could identify individual participants. All data were anonymized and de-identified prior to access.

### 2.2 Source of cases

Patients whose COVID-19 diagnosis was confirmed by polymerase chain reaction(PCR) testing of a nasopharyngeal sample were selected from the Second Hospital of Shanxi Medical University (2020–2022) via the Hospital Information System, and their general information, medical history, cardiac enzyme profiles, and imaging data were extracted for retrospective analysis.

### 2.3 Inclusion and exclusion criteria

The inclusion criteria were as follows: (1) meeting the criteria for confirmed COVID-19 patients; (2) age ≥ 18 years; (3) complete medical records; and (4) absence of concomitant organic diseases such as angina, acute myocardial infarction, myocarditis, cardiomyopathy, and heart failure. The exclusion criteria were as follows: (1) lack of willingness to participate in the study; (2) patients with incomplete clinical data; and (3) patients with arrhythmias triggered by emotional stress, vigorous exercise, excessive alcohol or coffee consumption, consumption of strong tea, or persistent smoking.

### 2.4 Methods

#### 2.4.1 Methods of routine electrocardiogram (ECG), dynamic electrocardiogram (DCG) and echocardiography.

ECG examination was performed using an electrocardiogram machine (MECG-200; MedEx Medical Technology Co., Ltd., Beijing, China) for 12-lead ECG examination. The patient was placed in a supine position, lying flat on the examination bed and kept relaxed. Fully expose the locations where leads need to be connected (chest, wrists of both upper limbs, ankles of both lower limbs) and apply conductive glue to connect the limb induction line and the chest induction line. The chest induction wire electrodes (to be used after disinfection) were respectively connected to V1 (the 4th intercostal space at the right margin of sternum), V2 (the 4th intercostal space at the left margin of sternum), V4 (the 5th intercostal space at the left midclavicular line), V3 (the middle of the V2 and V4 leads), V5 (the left anterior axillary line is level with V4), and V6 (the left midaxillary line is level with V4). The sensitivity was set at 10 mm/mV and the paper speed at 25 mm/s, and the routine ECG changes were recorded by the instrument. According to the detected Q wave, R wave, S wave and T wave, the doctor can determine whether the patient is accompanied by myocardial ischemia and arrhythmia.

DCG examination was performed by using a holter electrocardiogram (MIC-12H-3S; Beijing Shiji Jinke Medical Instrument Co., Ltd., Beijing, China). The patient was placed in a supine position, the chest skin was fully exposed to keep it relaxed, and the instrument was connected to the patient according to the above ECG method, with the same parameters as the ECG examination. After the holter ECG is correctly connected to the patient, the paper speed is set to 25 mm/s to ensure that the image is clear during the tracing process. To guide the patients to do appropriate exercise, so as to facilitate the medical staff to carry out continuous 24h ECG monitoring of the patients’ myocardial ischemia or arrhythmia. During the 24h ECG monitoring, large exercise, sweating, smoking, drinking and overwork should be avoided, and multi-position ECG of patients should be recorded as far as possible to reduce experimental errors. The doctor analyzed the electrocardiogram to determine whether there was myocardial ischemia or arrhythmia.

Echocardiography: Color Doppler ultrasound diagnostic instrument (EPIQ 7C; Philips, Amsterdam, The Netherlands), the patient was placed in the left lateral position, The medical staff applied gel to the ultrasound probe and placed it on the side of the sternum, apex of the heart, subcostal and section (such as long axis, short axis and four chambers) to collect indicators such as left atrial diameter (LAD), left ventricular end-diastolic diameter (LVEDD), end-systolic volume (ESV), end-diastolic volume (EDV), aortic root diameter and left ventricular ejection fraction (LVEF). LVEF was specifically quantified using the modified Simpson’s biplane method. In order to ensure the accuracy of the data, three consecutive measurements were required and the average value was taken.

#### 2.4.2 Myocardial enzyme profile, High-sensitivity troponin I (hs-TnI) and N-terminal pro B-type natriuretic peptide (NT-proBNP) detection.

Collect 3-5mL venous blood from patients on an empty stomach, centrifuge the supernatant (Speed: 3000 r/min, 15 min), and submit it for examination immediately. The levels of hs-TnI, NT-proBNP and myocardial enzyme (including lactate dehydrogenase (LDH), creatine kinase (CK), creatine kinase isoenzyme (CK-MB) and myoglobin were measured by automatic biochemistry analyzer (mini-VIDAS; Biomerieux, Marcy-l'Étoile, France) and automatic chemiluminescence immunoassay analyzer (ACCESS2; Beckman Coulter, Brea, CA, USA) and supporting kits.All tests are performed in strict accordance with the kit operating instructions to ensure the reliability of test results.

Hs-TnI determination: (1) Experimental principle: Quantitative measurement by chemiluminescence method. (2) Operation steps: ①Remove the kit from the refrigerator and balance it at room temperature for 30 minutes. ②Add the standard sample and the sample to be tested, and use a reagent strip for each sample and standard to be tested. ③Load the reagent strip into the ACCESS2 instrument. ④The reagent is put into the instrument to start the test, and all the analysis process is automatically completed by the instrument. ⑤All results are automatically generated by the instrument.

Determination of NT-proBNP: (1) Experimental principle: Quantitative determination by immunodouble antibody sandwich method and fluorescence detection method. (2) Operation steps: ①Remove the kit from the refrigerator and balance it at room temperature for 30 minutes. ②Add the standard sample and the sample to be tested. For each sample and standard to be tested, use one PBNP reagent strip and one PBNP SPR. ③Load the SPRs and reagent strips into the mini-VIDAS automatic analyzer. ④The reagent is put into the instrument to start the test, and all the analysis process is automatically completed by the instrument. ⑤ All results are automatically generated by the instrument.

### 2.5 Statistical analysis

Data processing and analysis were conducted using SPSS software (version 26.0) and R software (version 4.4.3). Normally distributed metric data are presented as mean ± standard deviation (SD), and group comparisons were performed using the t-test. Non-normally distributed metric data are presented as median and quartiles [M(P25, P75)], and group comparisons were performed using nonparametric tests. Count data are presented as percentages (%), and group comparisons were performed using the χ2 test. The risk factors for COVID-19 patients developing arrhythmia were evaluated using the univariate logistic regression analysis method. The independent risk factors influencing the occurrence of arrhythmia in COVID-19 patients were determined using multivariate logistic regression. The receiver operating characteristic (ROC) curve was constructed to assess the diagnostic performance of the regression equation for predicting arrhythmia in COVID-19 patients, as well as for obtaining the area under the curve (AUC), sensitivity, and specificity. All statistical tests were two-tailed, with *P* < 0.05 indicating statistical significance.

## 3. Results

### 3.1 General information

**Fig 1:**
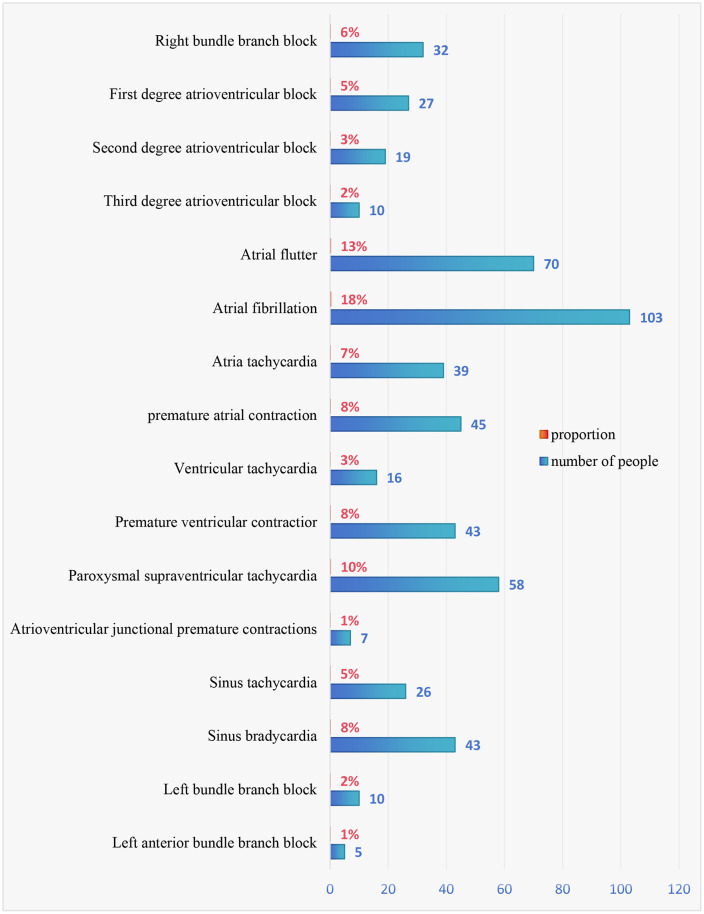
Types and rates of arrhythmias in COVID-19 patients.

A total of 324 confirmed COVID-19 patients from the Second Hospital of Shanxi Medical University from 2020 to 2022 were included, with ages ranging from 23 to 94 years with an average of 69 years; 150 were female and 174 were male. Of the patients, 15.4% had a history of diabetes, and 51.5% had a history of hypertension. Among the 324 COVID-19 patients included, 257 patients experienced arrhythmias (Fig 1).

### 3.2 Clinical characteristics of arrhythmias in COVID-19 Patients

Based on whether COVID-19 patients had arrhythmias, all patients were divided into arrhythmia and non-arrhythmia groups. There were statistically significant differences (*P* < 0.05) in heart rate, prothrombin time (PT), activated partial thromboplastin time (APTT), blood glucose, uric acid, serum potassium, total serum cholesterol, hs-TnI, NT-proBNP, aortic root diameter, LAD, LVEDD, LVEF, FS, ESV, and diabetes between the two groups. However, there were no statistically significant differences (*P* > 0.05) in age, gender, body temperature, systolic blood pressure (SBP), diastolic blood pressure (DBP), respiratory rate (RR), hemoglobin, white blood cell count, red blood cell count, lymphocyte count, monocyte count, lymphocyte percentage, neutrophil percentage, neutrophil count, Aspartate aminotransferase/Alanine aminotransferase (AST/ALT) ratio, creatinine, urea, serum albumin, serum calcium, serum sodium, serum chloride, serum triglycerides, CK, myoglobin, EDV, stroke volume (SV), length of hospitalization, D-dimer, C-reactive protein (CRP), procalcitonin (PCT), LDH, CK-MB and the presence of hypertension between the two groups (Table 1).

**Table 1:** Characteristics of arrhythmia and non-arrhythmia groups.

Variable		Arrhythmia Group (n = 257)	Non-arrhythmia Group (n = 67)	*P*
**Age (years)**		70 (62, 79.5)	72 (60, 80)	0.759
**Gender (n,%)**	**Male**	144(56.00)	30(44.80)	0.100
	**Female**	113(44.00)	37(55.20)	
**Body Temperature(°C)**		36.50 (36.20, 36.60)	36.50 (36.20, 36.60)	0.232
**HR(times/min)**		80 (70, 90)	75 (70, 82)	0.010
**SBP(mm Hg)**		125 (112, 136)	127 (120, 135)	0.139
**DBP(mm Hg)**		73 (65, 80)	74 (69, 84)	0.232
**RR(breaths/min)**		20.00 (20.00-20.00)	20.00 (19.50-20.00)	0.490
**D-Dimer(ng/mL)**		164.00 (71.00-456.00)	167.00(79.00-502.00)	0.972
**PT(s)**		14.30 (13.00-15.70)	13.50 (13.00-14.45)	0.006
**APTT(s)**		30.80 (28.50-33.70)	29.70 (27.60-32.25)	0.017
**CRP(mg/L)**		20.95 (5.88-53.31)	29.47 (3.66-48.09)	0.855
**PCT (ng/mL)**		1.13 (0.47-4.07)	1.16 (0.40-5.13)	0.449
**Hb (g/L)**		133.00 (120.00-148.00)	133.00 (117.50-146.50)	0.578
**White Blood Cell Count(×10^9/L)**		5.95 (4.75-7.74)	5.47 (4.48-7.72)	0.462
**Red Blood Cell Count(×10^12/L)**		4.34 (3.89-4.80)	4.26 (3.76-4.75)	0.407
**Lymphocyte Count(×10^9/L)**		1.24 (0.73-1.79)	1.24 (0.69-1.58)	0.599
**Monocyte Count (×10^9/L)**		0.42(0.32,0.56)	0.39(0.32,0.54)	0.182
**Lymphocyte Percentage (%)**		22.40 (11.70-30.80)	21.20 (12.50-29.40)	0.707
**Neutrophil Percentage (%)**		68.20 (58.80-79.70)	69.40 (60.35-79.70)	0.412
**Neutrophil Count(×10^9/L)**		3.89(2.90,5.38)	3.91(2.62,5.36)	0.901
**Blood Glucose(mmol/L)**		5.76 (5.02-7.21)	6.32 (5.61-7.96)	0.023
**AST/ALT**		1.30(0.98,1.76)	1.20 (0.91-1.67)	0.323
**Creatinine (umol/L)**		71.00 (59.00-89.00)	68.87 (58.52-77.00)	0.127
**Urea (mmol/L)**		5.69 (4.60-7.60)	5.81 (4.71-7.56)	0.988
**Uric Acid (umol/L)**		337.00 (270.00-429.00)	301.66 (230.25-386.06)	0.020
**Serum Albumin(g/L)**		37.40 (33.80-40.70)	37.40 (33.60-40.50)	0.685
**Serum Potassium (mmol/L)**		4.01(3.72,4.32)	3.87 (3.56-4.12)	0.025
**Serum Calcium (mmol/L)**		2.22 (2.12-2.32)	2.22(2.11,2.33)	0.922
**Serum Sodium (mmol/L)**		139.00 (136.00-141.00)	139.20 (135.55-141.00)	0.828
**Serum Chloride (mmol/L)**		104.80 (101.00-107.00)	105.00 (103.00-107.00)	0.661
**Serum Total Cholesterol (umol/L)**		4.04 (3.19-5.07)	4.41 (3.65-5.33)	0.013
**Serum Triglycerides(mmol/L)**		1.19 (0.88-1.74)	1.25 (0.98-1.74)	0.392
**hs-TnI(pg/mL)**		34.69 (5.31-1166.43)	11.02 (4.11-345.11)	0.024
**NT-proBNP(pg/mL)**		1530.13 (401.00-4299.44)	906.04 (215.84-3140.66)	0.038
**LDH(U/L)**		210.00 (170.00-277.00)	217.00 (152.65-282.40)	0.568
**CK(U/L)**		80.00 (55.00-140.00)	69.20 (45.50-166.91)	0.477
**CK-MB(U/L)**		9.60 (7.40-15.90)	9.80 (7.45-12.55)	0.497
**Myoglobin (ng/mL)**		39.90 (24.30-81.10)	37.70 (21.10-68.75)	0.504
**Aortic Root Diameter (mm)**		28.00 (26.00-30.00)	27.00 (25.00-29.00)	0.038
**LAD(mm)**		35.00 (31.00-40.00)	32.00 (29.00-36.00)	0.001
**LVEDD(mm)**		47.00 (45.00-51.00)	46.00 (44.00-49.00)	0.026
**LVEF(%)**		64.00 (60.00-68.00)	67.00 (63.00-70.50)	0.001
**FS (%)**		35.00 (32.00-38.00)	37.00 (33.50-39.00)	0.011
**ESV (mL)**		38.00 (31.00-50.00)	35.00 (28.00-43.00)	0.015
**EDV(mL)**		106.00 (89.00-128.00)	107.00 (92.00-124.50)	0.777
**SV(mL)**		69.00 (58.00-82.00)	71.00 (62.00-84.00)	0.258
**Length of hospitalization(days)**		9.00 (6.00-13.00)	8.00 (6.00-11.00)	0.436
**Diabetes(%)**	**Yes**	46(17.90)	4(6.00)	0.016
	**No**	211(82.10)	63(94.00)	
**Hypertension (%)**	**Yes**	135(52.50)	32(47.80)	0.487
	**No**	122(47.50)	35(52.20)	

**Note:** Normally distributed metric data are presented as mean ± standard deviation, and group comparisons were performed using the t-test; Non-normally distributed metric data are presented as median and quartiles [M(P25, P75)], and group comparisons were performed using nonparametric tests; Count data are presented as percentages (%), and group comparisons were performed using the χ2 test; RR, Respiratory Rate; HR, Heart Rate; SBP, Systolic Blood Pressure; DBP, Diastolic Blood Pressure; PT, prothrombin time; APTT, activated partial thromboplastin time; CRP, C-Reactive Protein; PCT, procalcitonin; Hb, Hemoglobin; AST/ALT, Aspartate aminotransferase/Alanine aminotransferase; hs-TnI, high-sensitivity troponin I; NT-proBNP, N-terminal pro B-type natriuretic peptide; LDH, lactate dehydrogenase; CK, creatine kinase; CK-MB, creatine kinase-MB; LAD, Left atrial diameter; LVEDD, Left ventricular end-diastolic diameter; LVEF, Left ventricular ejection fraction; FS, fractional shortening; ESV, end-systolic volume; EDV, end-diastolic volume; SV, stroke volume.

### 3.3 Univariate and multivariate logistic regression analysis of arrhythmias in COVID-19 patients

COVID-19 patients were classified according to whether they developed arrhythmias as the dependent variable, and all variables with statistically significant differences were included in a binary unconditional logistic regression analysis model. The univariate logistic regression analyses showed that heart rate, PT, APTT, uric acid, serum potassium, serum total cholesterol, hs-TnI, aortic root diameter, LAD, LVEDD, LVEF, FS, ESV and diabetes correlated with the presence of the arrhythmias in COVID-19 patients (Supplementary Table). The multivariate logistic regression analyses indicated that the heart rate, PT, hs-TnI, ESV, serum potassium, blood glucose and diabetes were independent risk factors for arrhythmias in COVID-19 patients (*P* < 0.05) (Table 2).

**Table 2:** Multivariate logistic regression analysis results of arrhythmia occurrence in COVID-19 patients (n = 324).

Factor	OR	95%CI	*P*
**PT**	1.164	1.020-1.359	0.038
**HR**	1.034	1.011−1.059	0.006
**ESV**	1.024	1.005−1.049	0.030
**Blood glucose**	0.793	0.706-0.886	<0.001
**hs-TnI**	1.000	1.000-1.001	0.034
**Serum potassium**	1.876	1.018-3.581	0.049
**Diabetes**	6.783	2.063-29.963	0.004
**AUC**	0.773 (0.711-0.834)		<0.001

**Note:** OR stands for odds ratio; 95% CI means 95% confidence interval. PT, prothrombin time; HR, Heart Rate; ESV, end-systolic volume; hs-TnI, high-sensitivity troponin I.

### 3.4 ROC Curve analysis

**Fig 2:**
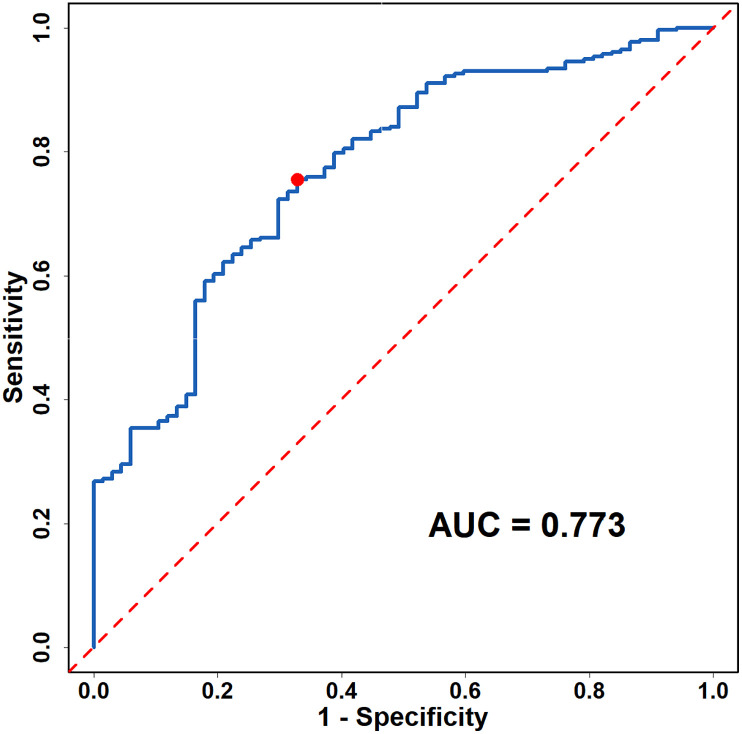
ROC curve of regression equation for assessing arrhythmia in patients with COVID-19.

ROC curve evaluation results indicated that the AUC was 0.773 (0.711–0.834), *P* < 0.001, the sensitivity was 0.755, and the specificity was 0.672 (Fig 2).

## 4. Discussion

Cardiac complications are a legacy of COVID-19, with patients exposed to the virus experiencing complications such as myocardial infarction, myocarditis and arrhythmias [6]. Of the 324 cases included in our study, 257 patients exhibited cardiac arrhythmias. Our study findings revealed that 73% of patients experienced tachyarrhythmias, with the rest experiencing bradyarrhythmias. Atrial arrhythmias were the most common type, with atrial fibrillation being the most common tachyarrhythmia at 18% and sinus bradycardia being the most common bradyarrhythmia at 8%. These results have also been confirmed in some studies. A study that involved 1,197 COVID-19 patients revealed that atrial fibrillation was the most common tachyarrhythmia (21%), whereas sinus bradycardia (8%) and complete heart block (8%) were the most common bradyarrhythmias [7]. The largest survey to date, which involved 4,526 hospitalized COVID-19 patients, reported that 18.27% of patients experienced arrhythmias caused by COVID-19. Of the arrhythmia cases, 22.6% were bradyarrhythmias, with the rest being tachyarrhythmias. Among the tachyarrhythmia cases, 81.8% were atrial arrhythmias, especially atrial fibrillation, atrial flutter, and supraventricular tachycardia, and 20.7% were ventricular arrhythmias [8]. Several studies [9–12] have also indicated an increased incidence of arrhythmias as cardiovascular complications of novel coronavirus infections. The patients with COVID-19 admitted to the intensive care unit had the higher arrhythmia rate, suggesting that the occurrence of cardiac arrest and arrhythmias may be related to the severity of the illness [10]. Overall, it has been reported that 10%–20% of COVID-19 patients develop arrhythmias, with the vast majority being curable with active management [13]. However, a small percentage of patients may experience various sequelae of arrhythmias.

Our study results on the occurrence of arrhythmia and risk factors for arrhythmia in COVID-19 patients with myocardial infarction indicated statistically significant differences between arrhythmia and non-arrhythmia patients in terms of heart rate, PT, APTT, blood glucose, uric acid, serum potassium, serum total cholesterol, hs-TnI, NT-proBNP, aortic root diameter, LAD, LVEDD, LVEF, FS, ESV, and diabetes. Multivariate logistic regression analysis revealed that heart rate, PT, hs-TnI, ESV, serum potassium, blood glucose and diabetes are risk factors for arrhythmia in COVID-19 patients. A retrospective study by Li et al. [14] on 785 patients demonstrated that SARS-CoV-2 exerts considerable cardiac impact, primarily manifested as elevated levels of NT-proBNP, D-dimer, CK, CK-MB, LDH, creatinine, and blood urea nitrogen. Huang et al. [15] observed that 12% of 41 COVID-19 patients exhibited reduced ejection fraction and elevated CK-MB levels.These patients also exhibited significantly elevated levels of hs-cTnI. Corroborating this, Cersosimo et al. [16] found that acute myocardial injury, indicated by elevated biomarkers such as NT-proBNP, CK-MB, and troponin, was associated with a dose-dependent increase in mortality risk and worsened prognosis. Furthermore, Yuniadi et al. [17] demonstrated that heart rate >85 bpm increased mortality risk in COVID-19 patients with arrhythmias. The findings of the present study align well with this established body of evidence. The underlying pathophysiology also appears to involve a systemic inflammatory response, as several studies [15, 18, 19] have reported significantly elevated inflammatory markers (including PCT, white blood cell count, and CRP) in patients with cardiac injury.

In our study, ROC curve evaluation results indicated that the combination of heart rate, PT, hs-TnI, ESV, serum potassium, blood glucose, and diabetes had moderate predictive performance for arrhythmia in COVID-19 patients (AUC = 0.773). This suggests that for COVID-19 patients, the early detection of risk factors is imperative. It may be necessary to conduct electrocardiograms, Holter monitoring, echocardiograms, and cardiac enzyme tests when required. Understanding the patient’s past cardiac history, defining the type of arrhythmia, assessing the risk of arrhythmia, and providing appropriate interventions and treatments are crucial. Considering the importance of cardiovascular diseases, especially arrhythmias, and their impact on the prognosis of COVID-19 patients, attention should be focused on psychological and social factors, underlying diseases, and the side effects of medications, as well as their effects on arrhythmias.

Various factors can cause arrhythmias triggered by the SARS-CoV-2 [20,21]. Based on current relevant clinical, laboratory studies, and autopsy results, there are several possible causes of arrhythmias. (1) Psychological and social factors [22]: With the changing situation of the COVID-19 epidemic, people are predisposed to feelings of tension, fear, and anxiety [23], especially among infected individuals. These physical and psychological stressors lead to the release of catecholamines, causing myocardial damage and subsequently resulting in cardiac arrhythmias. (2) Hypoxemia [24]: Respiratory distress is a common symptom in COVID-19 patients and can rapidly progress to acute respiratory distress syndrome, leading to significant gas exchange impairment, ultimately resulting in hypoxemia. Hypoxemia leads to increased metabolic demands and oxygen consumption, resulting in tachycardia, along with symptoms such as dyspnea, chest tightness, headache, and dizziness. In severe cases, it may lead to inadequate myocardial perfusion, causing complications such as arrhythmias and myocardial infarction. (3) Role of inflammatory factors: Inflammatory cell infiltration and cytokine release can directly cause myocardial injury. A study published in The Lancet revealed that many severe COVID-19 patients experience a “cytokine storm” [15]. (4) Role of angiotensin-converting enzyme 2 [25]: Angiotensin-converting enzyme 2 (ACE2) plays a crucial role in the heart, inhibiting myocardial fibrosis, reducing cardiac remodeling, improving heart function and vasodilation, and regulating blood pressure and fluid balance. It possesses anti-inflammatory and anti-proliferative effects. ACE2 has been identified as a functional receptor for SARS-CoV-2 [2,5]. Downregulation of ACE2 levels in COVID-19 patients can lead to or exacerbate cardiovascular diseases. (5) Association with preexisting cardiovascular diseases in patients: Clinical observations and research reports suggest that more than one-third of patients have comorbidities such as hypertension and cardiovascular diseases. Severe cases often occur in patients older than 70 years, with a higher probability of cardiovascular comorbidities. Therefore, in these patients, preexisting cardiovascular diseases and arrhythmias may be triggered by COVID-19 infection. (6) Adverse reactions to medications: Medications used to treat novel coronavirus infections may cause irregular heartbeats. For example, chloroquine has been adopted as one of the clinical treatments for COVID-19 infections, but in vitro results have confirmed that chloroquine can cause QT interval prolongation, conduction disorders, and enhanced automaticity. (7) Immune system reactions: After infection with the novel coronavirus, the immune system will produce a series of reactions, including the release of cytokines and antibodies, with a small amount of single-nuclear cell inflammatory infiltrates reaching the myocardial interstitium [26]. This may affect the heart and lead to arrhythmias. (8) Thrombosis: Novel coronavirus infection may lead to a prothrombotic state [27,28], increasing the risk of thrombosis and resulting in arrhythmias. (9) Fluid imbalance and electrolyte disorders: Patients with COVID-19 have downregulation of ACE2 receptors, reducing the feedback of ACE2 on the renin-angiotensin system (RAS) and subsequently leading to water and sodium retention and hypokalemia. Increased blood volume increases the heart’s preload, which can cause tachycardia. Hypokalemia can lead to metabolic abnormalities in myocardial cells, enhancing their automaticity and causing arrhythmias. (10) Endothelial dysfunction [29]: The novel coronavirus can directly damage endothelial cells, resulting in abnormal endothelial cell function. Endothelial dysfunction is associated with inflammation and clotting disorders, multiple organ damage, and eventual death in COVID-19 patients. It is important to note that arrhythmias may be a consequence of systemic disease and not just a direct effect of COVID-19 infection [10].

The heart is one of the most vulnerable target organs of the SARS-CoV-2, leading to a higher mortality rate for cardiovascular diseases, which is worrying. It is crucial to strengthen the monitoring of cardiac damage markers such as electrocardiograms [30], myocardial enzymes, troponin, and creatine kinase. In addition, the severity of COVID-19 is strongly associated with cardiac damage caused by infection with the SARS-CoV-2, Some similarities between the symptoms of COVID-19 infection and arrhythmia were also highlighted [31]. Therefore, clinicians should increase vigilance for signs of arrhythmias when treating patients with COVID-19 infection and actively monitor clinical symptoms, changes in relevant indicators, and potential adverse reactions from combined medication. Furthermore, there is a need to further explore different sites where the novel coronavirus affects the cardiovascular system to provide new insights for clinical treatment. In the study of cardiovascular system damage in COVID-19 patients, early studies mostly focused on specific mechanisms, related manifestations, and treatments. Thus, future studies should focus on long-term prognosis, understanding the disease’s developmental patterns, and intervening and improving adverse natural and treatment outcomes to enhance patients’ long-term quality of life. Psychological evaluation of patients should be conducted during treatment and prognosis, providing necessary psychosocial support therapy [32] to alleviate patients’ anxiety, fear, depression, and other negative emotions, thereby improving treatment outcomes and quality of life.

This study is distinct from previous research in that it includes a population of diabetic patients. This study found that COVID-19 patients with diabetes are more prone to developing arrhythmias. This suggests that in the clinical treatment of COVID-19 patients with arrhythmias, special attention should be focused on this group of patients, closely monitoring them, detecting and diagnosing early, and initiating treatment promptly to avoid worsening conditions.

Limitations: The data in this study came from patients in a single hospital center, which may have selection bias. In addition, some cases were limited by the pandemic conditions, preventing patients from completing tests such as electrocardiograms, echocardiograms, and cardiac biomarker assessments, which resulted in incomplete clinical data and a relatively small sample size. Furthermore, this study was retrospective and cross-sectional, thus warranting a larger sample size and high-quality prospective multicenter studies on the risk factors for arrhythmias in COVID-19 patients. It is worth noting that over time, the pathogen of COVID-19, the SARS-CoV-2 continues to evolve and mutate, giving rise to various mutated strains with changes in transmissibility and virulence, examples include Alpha (B.1.1.7), Beta (B.1.351), Gamma (P.1), Delta (B.1.617.2), and Omicron (B.1.1.529). In the context of the global pandemic, the emergence of mutated strains poses a huge threat to the COVID-19 response. Therefore, in future studies, whether there is a correlation between different COVID-19 variants and the incidence of arrhythmia is really worth our in-depth consideration.

## 5. Conclusions

In summary, we found that the heart rate, PT, APTT, blood glucose, uric acid, serum potassium, serum total cholesterol, hs-TnI, NT-proBNP, aortic root diameter, LAD, LVEDD, LVEF, FS, ESV, and the presence of diabetes have significant differences in COVID-19 patients with arrhythmia compared with COVID-19 patients without arrhythmia. Heart rate, PT, hs-TnI, ESV, serum potassium, blood glucose and diabetes are risk factors for arrhythmia in COVID-19 patients.This indicates that the glycemic control and cardiac function improvement can effectively reduce the risk of arrhythmia. These findings provide a basis for developing targeted treatment strategies for COVID-19 patients complicated with arrhythmia, while also offering important guidance for preventive measures, thereby contributing to improved quality of life. Future studies should focus on long-term prognosis evaluation and further optimization of individualized intervention protocols.

## Supporting information

S1 Table Univariate logistic regression analysis results of arrhythmia occurrence in COVID-19 patients (n = 324). (DOCX)
