# Supplementary material for: Analysis of arrhythmia and its risk factors in patients with COVID-19
Source: PLoS One. 2025 Nov 25;20(11):e0336370. doi: 10.1371/journal.pone.0336370 (PMC12646396; doi:10.1371/journal.pone.0336370)
Supplement: S1 Table — (DOCX) [file pone.0336370.s001.docx]

Supplementary Table. Univariate logistic regression analysis results of arrhythmia occurrence in COVID-19 patients (n=324).

| **Factor** | **OR** | **95%CI** |  | ***P*** | | | |  | |
| --- | --- | --- | --- | --- | --- | --- | --- | --- | --- |
| **LVEF** | 0.939 | 0.898-0.977 |  | 0.003 | | | |  | |
| **PT** | 1.217 | 1.072-1.406 |  | 0.005 | | | |  | |
| **LAD** | 1.058 | 1.019-1.102 |  | 0.005 | | | |  | |
| **ESV** | 1.026 | 1.009-1.047 |  | 0.007 | | | |  | |
| **HR** | 1.025 | 1.006-1.046 |  | 0.011 | | | |  | |
| **FS** | 0.933 | 0.882-0.984 |  | 0.013 | | | |  | |
| **Uric** Acid | 1.003 | 1.001-1.005 |  | 0.020 | | | |  | |
| **hs**-TnI | 1.000 | 1.000-1.001 |  | 0.020 | | | |  | |
| **LVEDD** | 1.065 | 1.012-1.126 |  | 0.021 | | | |  | |
| **Serum** Total Cholesterol | 0.778 | 0.626-0.964 |  | 0.022 | | | |  | |
| **Serum Potassium** | 1.909 | 1.104-3.366 |  | 0.022 | | | |  | |
| **Diabetes** | 3.434 | 1.330-11.709 |  | 0.023 | | | |  | |
| **Aortic** Root Diameter | 1.100 | 1.014-1.199 |  | | 0.024 |  |  |  |  |
| **APTT** | 1.058 | 1.008-1.122 |  | | 0.038 | | |  |  |
| **NT**-proBNP | 1.000 | 1.000-1.000 |  | | 0.051 | | |  |  |
| **CK**-MB | 1.029 | 1.001-1.067 |  | | 0.082 | | |  |  |
| **Gender** | 1.572 | 0.917-2.713 |  | | 0.101 | | |  |  |
| **Monocyte** Count | 2.738 | 0.786-12.073 |  | | 0.159 | | |  |  |
| **Blood Glucose** | 0.947 | 0.878-1.025 |  | | 0.160 | | |  |  |
| **Creatinine** | 1.006 | 0.999-1.015 |  | | 0.161 | | |  |  |
| **Urea** | 1.044 | 0.986-1.126 |  | | 0.197 | | |  |  |
| **Body Temperature** | 0.681 | 0.376-1.269 |  | | 0.207 | | |  |  |
| **DBP** | 0.988 | 0.967-1.009 |  | | 0.244 | | |  |  |
| **Myoglobin** | 1.001 | 1.000-1.004 |  | | 0.253 | | |  |  |
| **Serum** Albumin | 1.025 | 0.980-1.073 |  | | 0.279 | | |  |  |
| **LDH** | 1.002 | 0.999-1.004 |  | | 0.290 | | |  |  |
| **SV** | 0.992 | 0.978-1.007 |  | | 0.306 | | |  |  |
| **D**-Dimer | 1.000 | 1.000-1.001 |  | | 0.315 | | |  |  |
| **CK** | 1.000 | 0.999-1.000 |  | | 0.320 | | |  |  |
| **PCT** | 1.029 | 0.982-1.102 |  | | 0.321 | | |  |  |
| **Length** of Hospitalization | 1.024 | 0.978-1.078 |  | | 0.333 | | |  |  |
| **Serum Calcium** | 1.313 | 1.012-2.766 |  | | 0.405 | | |  |  |
| **RR** | 1.055 | 0.938-1.215 |  | | 0.413 | | |  |  |
| **CRP** | 1.003 | 0.996-1.010 |  | | 0.441 | | |  |  |
| **Red Blood Cell** Count | 1.142 | 0.811-1.612 |  | | 0.447 | | |  |  |
| **Hypertension** | 1.210 | 0.706-2.080 |  | | 0.487 | | |  |  |
| **Neutrophil** Percentage | 0.994 | 0.975-1.013 |  | | 0.513 | | |  |  |
| **EDV** | 1.002 | 0.995-1.010 |  | | 0.529 | | |  |  |
| **SBP** | 0.996 | 0.985-1.009 |  | | 0.538 | | |  |  |
| **Lymphocyte** Count | 1.077 | 0.842-1.484 |  | | 0.605 | | |  |  |
| **Serum Chloride** | 0.988 | 0.942-1.033 |  | | 0.609 | | |  |  |
| **Neutrophil** Count | 0.978 | 0.893-1.081 |  | | 0.650 | | |  |  |
| **Serum Sodium** | 0.992 | 0.948-1.021 |  | | 0.679 | | |  |  |
| **White Blood Cell** Count | 1.009 | 0.923-1.112 |  | | 0.846 | | |  |  |
| **Lymphocyte** Percentage | 1.002 | 0.981-1.024 |  | | 0.849 | | |  |  |
| **Hb** | 1.001 | 0.989-1.012 |  | | 0.880 | | |  |  |
| **AST**/ALT | 1.017 | 0.819-1.347 |  | | 0.890 | | |  |  |
| **Age** | 1.001 | 0.982-1.020 |  | | 0.899 | | |  |  |
| **Serum** Triglycerides | 1.002 | 0.747-1.391 |  | | 0.990 | | |  |  |
|  |  |  |  | |  | | |  |  |

**Note:** OR stands for odds ratio; 95% CI means 95% confidence interval. RR, Respiratory Rate; HR, Heart Rate; SBP, Systolic Blood Pressure; DBP, Diastolic Blood Pressure; PT, prothrombin time; APTT, activated partial thromboplastin time; CRP, C-Reactive Protein; PCT, procalcitonin; Hb, Hemoglobin; AST/ALT, Aspartate aminotransferase/Alanine aminotransferase; hs-TnI, high-sensitivity troponin I; NT-proBNP, N-terminal pro B-type natriuretic peptide; LDH, lactate dehydrogenase; CK, creatine kinase; CK-MB, creatine kinase-MB; LAD, Left atrial diameter; LVEDD, Left ventricular end-diastolic diameter; LVEF, Left ventricular ejection fraction; FS, fractional shortening; ESV, end-systolic volume; EDV, end-diastolic volume; SV, stroke volume.
